# Rapid dopaminergic signatures in movement: Reach vigor reflects reward prediction error and learned expectation

**DOI:** 10.1126/sciadv.adz9361

**Published:** 2026-02-27

**Authors:** Colin C. Korbisch, Alaa A. Ahmed

**Affiliations:** ^1^ Department of Mechanical Engineering, University of Colorado Boulder, Boulder, CO, USA.; ^2^ Biomedical Engineering Program, University of Colorado Boulder, Boulder, CO, USA.

## Abstract

Movements become more vigorous when rewards are expected, suggesting that motivational signals influence motor control. Dopaminergic neurons, known to encode reward expectation and prediction error, are prime candidates for linking value and vigor. Here, we show that human reach vigor dynamically tracks canonical dopaminergic learning signals not only at movement onset but also during ongoing motion. Using a reaching task with probabilistic rewards (0, 33, 66, and 100%), we observed that peak velocity scaled with expected value. Crucially, following feedback, reach velocity was transiently invigorated or enervated in proportion to the sign and magnitude of the reward prediction error. Trial-by-trial changes in kinematics reflected value updating, consistent with dopaminergic phasic learning signals. These results demonstrate that movement vigor is modulated by reward-learning signals on rapid timescales, revealing a real-time behavioral readout of motivational computation in the brain.

## Teaser

Movement vigor rapidly tracks neural signatures of reward prediction error.

## INTRODUCTION

Individuals move faster, or with greater vigor, when pursuing more valuable goals (*1*–*10*). They are willing to produce greater muscle forces, or expend greater effort, to accomplish a movement aim in less time. A potential explanation for how this interaction may be mediated in the brain lies in the neurotransmitter dopamine (DA), which is implicated in both the learning and representation of value and the control of movement (*11*). Basal ganglia activity, which is already known to be influenced by dopaminergic inputs, has been found to invigorate not only movement vigor (*12*–*15*) but also the decision-making process (*16*, *17*). If vigor is a reflection of value and DA is the mediator, then vigor may also reflect the machinery that underlies the learning process, due to the phasic DA release coincident with learning and reward prediction error (RPE) (*18*). Seminal work has shown that dopaminergic neuron (DAN) responses in a learned environment scale with RPE at the time of both stimulus presentation and feedback presentation (*19*). DAN activity is greater in response to cues associated with greater expectation of reward (greater positive RPE) and lower upon feedback of that reward (lower positive RPE). If a similar time-locked response is present in movement vigor at cue and feedback presentation, then this would suggest that DA-related activity contributes to reward-driven increases in vigor.

While short-term phasic DAN response is implicated in prediction error and learning, tonic DA levels have been found to be sensitive to the history of reward reception (*1*, *20*–*22*). Particularly, midbrain DA levels approximate average reward rate and are implicated in motivation and invigoration. Hamid and colleagues found a strong relationship between history of reward and relative DA in the nucleus accumbens (Nac). Greater tonic levels of DA can also be interpreted as greater motivation or drive, resulting in behavioral invigoration (*23*, *24*). Recent reward history can influence movement vigor (*20*, *25*–*28*).

A growing body of work has revealed correlations between phasic dopaminergic activity and movement vigor (*29*–*37*). Cue-evoked phasic DA response within rat NAc core was significantly correlated with trial movement vigor (*30*). Similarly, for rat substantia nigra pars compacta (SNc) neurons sensitive to free movement initiation, relative activity levels were correlated with ensuing vigor (*34*). Optogenetic activation of these neurons when not moving increased the probability of movement initiation but did not produce any measurable changes in ongoing movement vigor. However, a separate study revealed that ongoing movement vigor was reduced with the inhibition of striatal activity (*33*). These and other works provide evidence that not only do longer timescale tonic DA levels potentially modulate animal movement vigor, so, too, may short latency, phasic responses.

In this study, we asked whether human reach kinematics reflect canonical features of dopaminergic reward signaling, including (i) expectation-dependent invigoration, (ii) short-latency modulation by RPE, and (iii) trial-by-trial adaptation reflecting learned value and reward history. To test these hypotheses, we designed a reaching task with probabilistic rewards to elicit known dopaminergic signatures related to expectation and prediction error. We found that movement vigor increased with reward expectation and that velocity was rapidly modulated after outcome feedback in accordance with signed RPE. In addition, kinematic responses reflected trial-wise value updating as well as longer-term reward history. These results highlight how motor planning, execution, and feedback control are all likely influenced by DA response, reflecting DA’s tripartite role in learning, motivation, and movement control.

## RESULTS

**Fig. 1.**
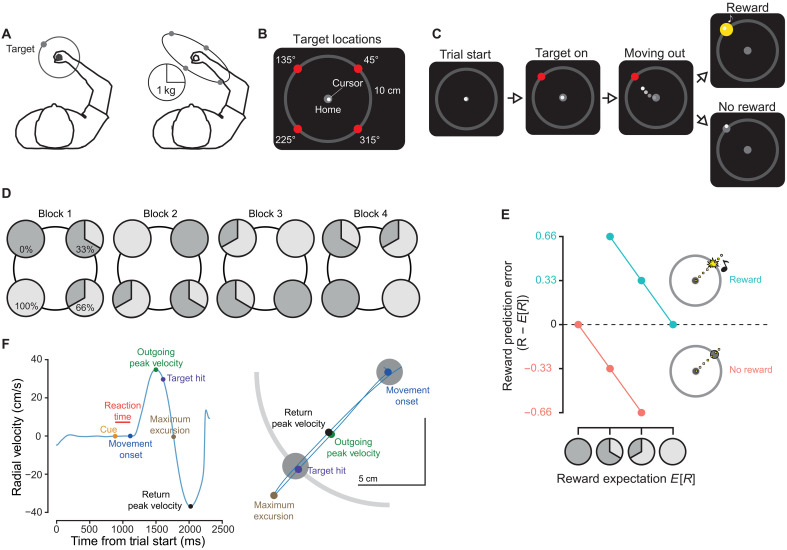
Experimental protocol. (**A**) Left: Participants made out-and-back reaches toward prompted, virtual targets. Right: Biomechanical inertia ellipse, centered on the hand. Based on participants’ posture during the reaching task, some directions had greater inertia compared to others. (**B**) Targets were located at four different positions, each 10 cm from a central location. (**C**) After the target was cued, participants were to make a reach toward said target. Reward feedback was dependent on target reward frequency, with reward feedback consisting of a yellow flash and high-pitched tone. Omission of feedback coincided with the target turning gray. (**D**) At the beginning of each 180-trial block, in the first experiment, participants were told the reward probabilities of each target: 0, 33, 66, or 100%. Relative order of reward-target associations was randomized across participants. (**E**) RPE was defined as the difference between the stated (and actual) reward frequency per target and the binary outcome of the trial. (**F**) Left: Kinematic measures of interest reaction time with example of trial reach trajectory given on the right.

In a series of two experiments, we asked whether movement vigor tracked canonical determinants of DA-related learning and performance: learned value, RPE, and reward history. In both experiments, human subjects directed arm reaching movements to targets associated with a probability of receiving a reward and differing biomechanical inertia (Fig. 1, A to C; see Materials and Methods). Each of the four target locations was associated with a unique probability of receiving the reward (0, 33.$\bar{3}$, 66.$\bar{6}$, and 100%) that was changed each block (Fig. 1D) of 180 trials. Feedback of reward reception or reward denial was provided upon target acquisition. Assuming perfect representation of task instruction, participants could experience five RPEs: −0.33 and +0.66 for the 66% target, −0.66 and 0.33 for the 33% target, and 0 for the 0 and 100% targets (Fig. 1E). This paradigm allowed us to probe the effect of reward expectation on the vigor of the outgoing movement. In addition, we could investigate the effect of RPE at time of feedback on the vigor of the return movement. Last, we tested the effect of reward history on reach vigor across trials, independent of the expected value of the current target. We predicted that, with increasing expectation, outgoing vigor would increase, reflected in an increase in peak velocity (Fig. 1F); that vigor would be modulated on the return portion of the reach, scaled by the RPE; and that vigor across trials would increase with reward history.

### Experiment 1

In the first experiment, we focused solely on the vigor response to a known expectation of reward (*E*[*R*]). We did so by informing the participants of the reward probability of the targets at the beginning of each block.

#### Peak velocity to target tracks reward expectation

**Fig. 2.**
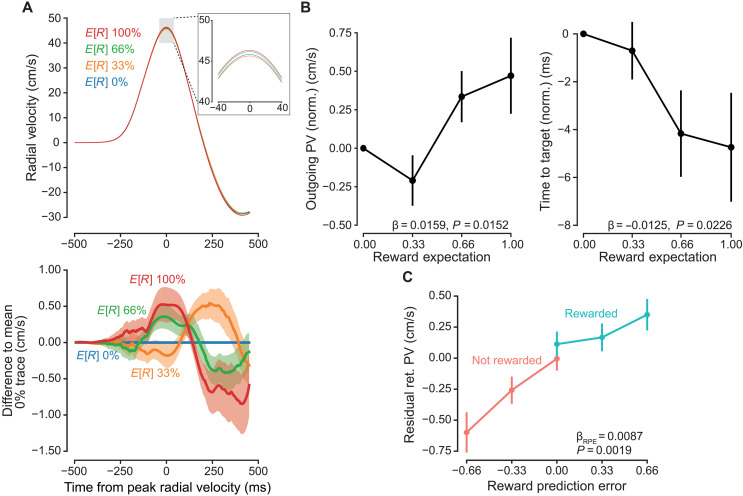
Vigor tracks reward expectation. (**A**) Top: Radial velocity trace for first experiment, aligned to time of outgoing peak velocity. Highlighted region shows differences in peak excursion velocity for differences in reward expectations. Bottom: To calculate the difference traces, each participant’s average signed radial velocity, at each sampled time point for the 0% expected reward condition, was subtracted from all other trials. Traces shown are the grand average of these difference traces, ±SEM. (**B**) Left: Outgoing peak velocity (PV) increased with increasing expected reward. Data were normalized by subtracting the per-participant average for 0% expected reward condition. Right: Time to target hit, defined as the time between movement onset and reaching out to a 10-cm radial distance, decreased as reward expectation increased. (**C**) Return peak velocities (ret. PV) were residualized by first fitting a reduced model including target direction and outgoing peak velocity. These residuals were significantly correlated with trial RPE, increasing with greater RPE. Error bars represent ±SEM.

As the cued target’s *E*[*R*] increased, peak velocity of the outgoing movement also increased [generalized linear mixed model (GLMM); β_*E*[*R*]_ = 0.0159 ± 0.00629, *P* = 0.0152; Fig. 2, A and B]. Consequently, time to target, defined as the time between movement onset and reaching a 10-cm radial distance, decreased with increasing reward expectation (β_*E*[*R*]_ = −0.0125 ± 0.00527, *P* = 0.0226). Despite the increased velocity associated with greater reward expectation, reach crossing point accuracy, calculated as unsigned angular error, was not influenced by reward expectation (*P* = 0.968; see the Supplementary Materials). Reaction times decreased with increasing *E*[*R*] (fig. S1, A and B).

#### Return velocity is influenced by RPE

When the cursor hits the target, participants can either receive reward feedback or not. Does this feedback, or lack thereof, influence subsequent movement kinematics? Further, does the degree of surprise also modulate movement kinematics? In other words, we asked whether RPE influenced the speed of the ensuing movement.

To answer these questions, we analyzed participants’ return movements and whether receiving (or not receiving) reward feedback on a given trial produced significant influences. Specifically, we questioned whether the sign and magnitude of the RPE influenced the velocity

RPE=R−E[R]

where R is a binary coding for the presence or absence of reward feedback (0, 1) and E[R] is the instructed expectation of reward feedback (see Materials and Methods).

Participants exhibited diminished peak velocity on the return movements compared to that on the outgoing movements (paired mean difference = 0.0601 m/s [0.0445, 0.0757 95% confidence interval], *t*_41_ = 7.7789, *P* = 1.34 × 10^−9^). After controlling for the average relationship between outward and return peak velocities (β_OutPV_ = 0.580 ± 0.0192, *P* < 2 × 10^−16^), we observed an effect of reward feedback and reward expectation, i.e., RPE (β_RPE_ = 0.00865 ± 0.00260, *P* = 0.00186; Fig. 2C). This indicated that the return portion of the reach was driven not only by feedforward mechanisms but also by feedback. No significant main effect of reward (β_Reward_ = 0.000876 ± 0.00159, *P* = 0.586) or interaction with RPE (β_RPE×Reward_ = −0.00545 ± 0.00392, *P* = 0.171) was found, indicating a slope of response across prediction error values without discontinuities. Additional control analyses confirmed that return movements were modulated by RPE, not by other potential confounds (figs. S2 to S4).

**Fig. 3.**
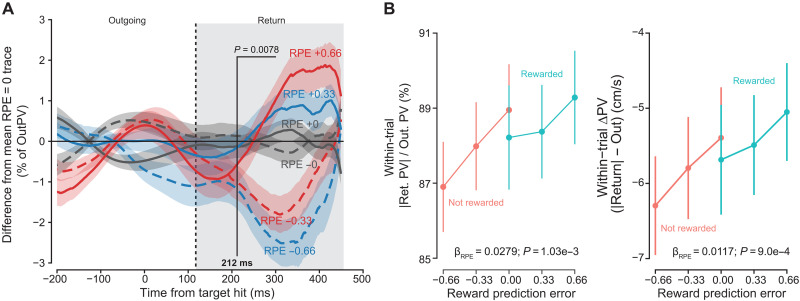
Vigor response reflects RPE. (**A**) For a within-trial measure of relative invigoration, instantaneous velocity was normalized by dividing by the trial outgoing peak velocity (OutPV). Difference traces were then calculated in a manner similar to Fig. 2A. From SPM analysis, a significant effect of RPE on percent peak velocity was found beginning at 212 ms after reward feedback was received. (**B**) The within-trial difference in absolute peak velocities. Left: Return divided by outgoing. Right: Return minus outgoing, significantly varied with RPE. As RPE increased, the return peak more closely matched the outgoing peak. Points are the grand mean and error bars, and shaded regions represent ±SEM.

To better account for variance in the outgoing portion of the movement, we normalized instantaneous velocity and focused on within-trial effects. First, instantaneous radial velocity was divided by the within-trial outgoing peak velocity. Next, the difference traces of these %-outgoing velocity values were calculated in a similar manner as in Fig. 2A, although, instead of taking the difference from the 0% reward trials, the differences were taken relative to the per-participant averaged normalized velocity trace for all 0 and 100% reward trials, i.e., RPE = 0 trials. After calculating the grand average of these difference traces, we observe a clear striation in relative normalized return velocity corresponding to the RPE (Fig. 3A).

We next performed a hierarchical random-effects analysis (fig. S5; see Materials and Methods) to determine the population-average effect of RPE on instantaneous, normalized velocity. At 212 ms after target feedback, we found a significant negative effect of RPE, indicative of greater invigoration in the return movement with greater RPE.

Aside from the effect on instantaneous velocity, the within-trial difference in velocities, i.e., the difference between excursion and return peak velocity, also exhibited a significant effect of RPE (Fig. 3B), with the slope of response consistent across positive and negative RPE conditions (β_RPE×Reward_ = −0.00176 ± 0.00502, *P* = 0.727). For both percent relative to outgoing peak (Fig. 3B, left) and velocity difference (Fig. 3B, right), the slope of response was significant across RPE conditions (β_RPE_ = 0.02788 ± 0.00788, *P* = 1.03 × 10^−3^; and β_RPE_ = 0.0117 ± 0.00325, *P* = 9.00 × 10^−4^, respectively). When RPE was most positive (+0.66), the difference between excursion and return peak velocity was at its smallest compared to RPE at its most negative (−0.66). There was no separate effect of reward feedback in either metric (β_Reward_ = −0.00269 ± 0.00463, *P* = 0.5643; and β_Reward_ = −0.00355 ± 0.00208, *P* = 0.0978).

Overall, we found a significant effect of reward feedback on the return movement that was dependent on the reward expectation, i.e., the RPE. With greater RPE, relative return velocity was greater than when the prediction error was negative. This difference emerged 212 ms after feedback was received and could not be accounted for by variation in maximum excursion or preemptive differences in the outgoing portion of the movement (potential correlations between outgoing peak velocity and future reward reception). Participants used neither set return velocity nor set return velocity difference strategies. In the first case, outgoing peak velocity would be unrelated to the return peak, and, in the second case, return velocity difference would be invariant to the experienced prediction error.

### Experiment 2

**Fig. 4.**
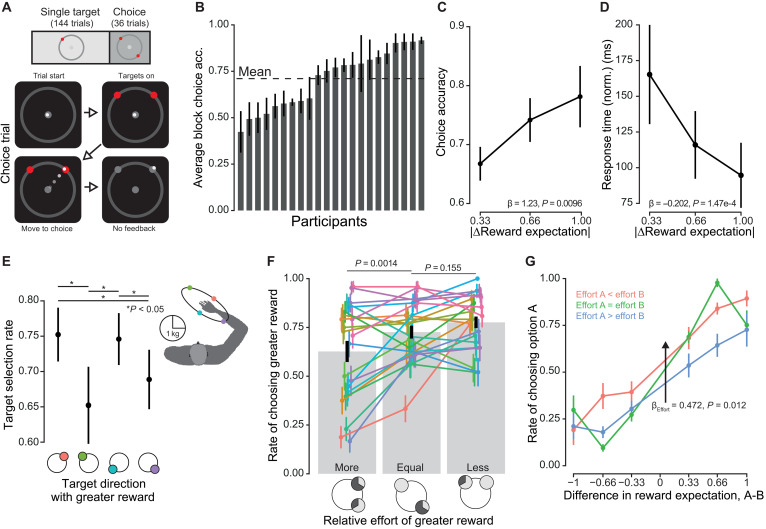
Experiment 2: Choice behavior. (**A**) In the second experiment, reward probabilities were left unstated to the participants. Instead, they were incentivized to learn the underlying rewards during the single-target period, as additional monetary compensation was dependent on performance during later choice trials. (**B**) Per-participant average accuracy rate over four blocks. Participant choices were highly variable across the population. Mean accuracy was 71%. (**C**) Participant choices were found to be sensitive to relative expectation, choosing the more rewarding option as difference increased. (**D**) Response time decreased with increasing difference of reward expectations of the presented targets. (**E**) Target direction affected choice selection. When targets 1 or 3 were the more rewarding option, i.e., associated with lesser biomechanical effort, they were selected at higher rates compared to when targets 2 or 4 were the more rewarding option, consistent with a potential influence of biomechanical effort on choice selection. (**F**) When grouping choices by whether the greater reward has greater relative effective mass, significant differences in choice accuracies were found. Individual participant responses are shown in color. (**G**) Baseline selection rates were shifted depending on relative effort. No interaction between effort and reward difference was found. Points are the grand mean, and error bars are ±SEM.

We next asked whether vigor was modulated by learned value rather than instructed value. To probe this, we conducted a second experiment in which participants were left uninformed of the targets’ reward probabilities. Instead, participants learned through experience over the course of a block, affording us a measure of trial-to-trial response of changes in reward estimation. Within each block, after a sequence of 144 single-target trials similar to those in the first experiment, 36 two-alternative forced choice trials were presented to assess degree of learning (Fig. 4A). Individuals were incentivized to choose greater expected rewards on choice trials as additional monetary compensation was dependent on performance (see Materials and Methods). Critically, no feedback was given on target hit during these choice trials to limit learning to the single-target trials only.

#### Choices reflected both expectation of reward and effort

Choice accuracy, defined as the selection of the greater reward probability of the two options, varied across the participants, ranging from 42.7 to 91.7% (Fig. 4B). The population-average accuracy rate was 71%. Underlying reward difference, hidden to participants, was found to significantly predict the rate of choosing one option over another (β_Δ*E*[*R*]_ = 1.233 ± 0.476, *P* = 0.0096; Fig. 4C). Response time of the decision significantly decreased with increasing differences in reward expectations (Fig. 4D; β_|Δ*E*[*R*]|_ = −0.202 ± 0.053, *P* = 1.47 × 10^−4^), providing further evidence that the underlying expectations were learned. Response time and outgoing peak velocity response differed during choice trials in their sensitivity to reward difference and choice accuracy (fig. S6). Whereas outgoing peak velocity only varied with the selected option’s reward expectation, response time varied with both relative and selected expectations.

Target direction was also found to influence decision-making. We first categorized all choices by whether a given target direction was associated with the greater of the two potential rewards presented on a given choice trial. We then calculated the choice accuracy rate for each of these subsets. If direction had no influence, then we should expect no variation in selection rates between these four categories; however, this was not the case. Instead, significant differences were found (Fig. 4E). Selection rate biases matched the arm’s inertial axes (Fig. 4E, right) (*38*, *39*). Using this measure of effort, we categorized choice trials by whether the more rewarding option was associated with more or less effort compared to the alternative choice (Fig. 4F). We found significant differences in accuracy rates, with idiosyncratic responses to effort readily apparent. Augmenting our logistic regression analysis from before by including a relative effort term, we found a significant effect of said relative effort, with average rates of choosing the more rewarding option increasing when this option was associated with less effort (β_e_ = 0.472 ± 0.188, *P* = 0.012; Fig. 4G). In addition, we found a significant correlation between reward and effort sensitivity [Pearson’s correlation, *t*_20_ = 2.1557, correlation coefficient (*r*) = 0.434, *P* = 0.04346; fig. S7]. Thus, participants’ choices revealed that they had largely learned the target reward contingencies and demonstrated that these value-based choices were influenced by the effort of the arm reach.

#### Velocity in single-target trials tracked reward expectation and reflected learning

**Fig. 5.**
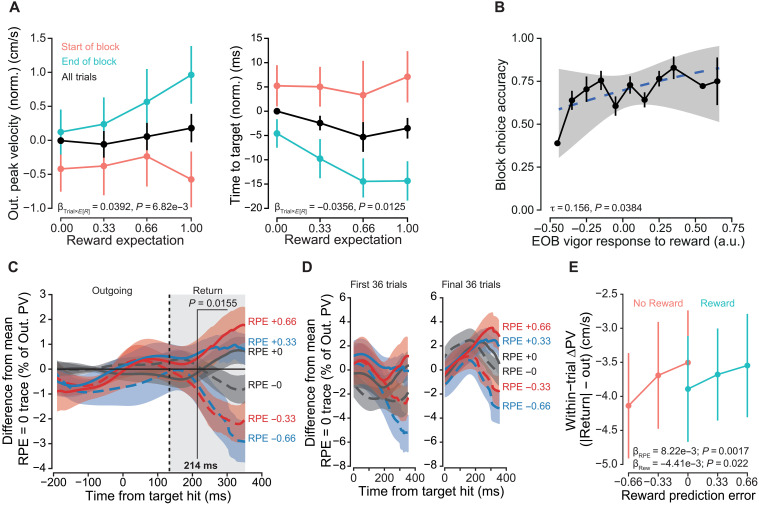
Experiment 2: Vigor tracked reward expectation and RPE. (**A**) Left: Outgoing peak velocity increased with increasing reward expectation. Data were normalized by taking the difference to per-participant average for 0% reward. The slope of peak velocity response to reward expectation changed over the course of a block. In the initial 36 trials (start of block), response was essentially flat, but, by the final 36 trials (end of block), outgoing velocity significantly increased with respect to reward frequency. Right: Time to target, taken as the duration between movement onset and reaching out to a 10-cm radius (target hit) changed with respect to underlying expectation over the course of the single-target period. (**B**) End of block response to reward (i.e., per-participant slope of response to *E*[*R*]) was significantly correlated with block choice accuracy (rate of choosing the more rewarding option, Fig. 4B). Dotted line and shading are model estimate and SD. a.u., arbitrary unit. (**C**) Normalized velocity difference traces for experiment 2. Data presented are the grand average of these difference traces. Differences in relative velocity emerged after reward feedback that scaled with prediction error. (**D**) We separated these traces for the start (first 36 trials) and end (last 36 trials) of the single-target period, demonstrating the shift in response striations. Out. PV, outgoing peak velocity. (**E**) As RPE increased, the relative reduction in peak velocities between the return and outgoing portions of the reaching movement decreased (i.e., the difference was less negative). Points represent grand means, and error bars and shaded regions represent ±SEM.

Given that participants’ choices revealed a learned reward estimation, we asked whether vigor on single-target trials would reveal this estimation as learning progressed. Over the course of the single-target period, we found that average slope of response of outgoing peak velocity relative to reward expectation increased (GLMM; β_Trial×*E*[*R*]_ = 0.0392 ± 0.01447, *P* = 0.00682), demonstrating a dynamic response to the probabilistic reward (Fig. 5A). Likewise, the effect of expectation on the time to target significantly varied over this period (β_Trial×*E*[*R*]_ = −0.0356 ± 0.0142, *P* = 0.0125). At the beginning of a block, there was no significant effect of reward expectation (β_*E*[*R*]_ = −0.0108 ± 0.0073, *P* = 0.142). The change in response of instantaneous velocity over the course of the single-target period is further evident in the velocity difference traces (fig. S8). However, no such interaction effect between trial and hidden reward expectation was found when modeling reaction times (fig. S1). As in experiment 1, we found no effect of reward expectation on reach crossing point accuracy (*P* = 0.232; see the Supplementary Materials).

The strength of peak velocity-reward expectation response (i.e., the slope of velocity relative to reward) could predict within-block choice accuracy (the rate of choosing the more rewarding of the two options). Across the participant pool, we found a significant correlation between these measures (Kendall’s rank correlation, τ = 0.156, *P* = 0.0384; Fig. 5B). The stronger the slope of response in an individual at the end of the single-target period, the greater the rate in selecting the more rewarding of the two options presented during choice trials.

Even when participants were not told of the expected reward probabilities at the outset of a block, both outgoing peak velocity and time to target responded to increasing expectation. Learning of the different reward frequencies is shown clearly by the differential response of velocity at the beginning and end of the single-target period. Initially, kinematic response did not differ between the four targets, but, by the end, average peak velocity increased with increasing expectation of reward feedback. Within-block performance could be predicted on the basis of participants’ slope of response to reward at the end of the single-target period, suggesting that the change in kinematics over the course of a block was related to the learning of relative reward frequencies.

#### Change in return velocity tracks RPE

Turning to within-trial measures of relative vigor, we performed the same hierarchical random effect analysis on normalized velocity as in the first experiment and found a population-level effect of RPE (Fig. 5C). Additional supporting details for this analysis are provided in the Supplementary Materials (fig. S9). At 214 ms after reward feedback, relative velocity varied significantly with RPE. Qualitatively, we observe the change in kinematic response of instantaneous velocity over the course of the single-target period (Fig. 5D). The within-trial velocity difference (return minus outgoing) also significantly varied with RPE (β_RPE_ = 8.22 × 10^−3^ ± 2.61 × 10^−3^, *P* = 0.00166; Fig. 5E). With increasing positive prediction error, the return peak velocity more closely matched the outgoing peak velocity. Reward feedback had no significant interaction effect on this difference (β_RPE×Reward_ = −2.156 × 10^−3^ ± 3.682 × 10^−3^, *P* = 0.558), However, there was a significant main effect of reward (β_Reward_ = −4.414 × 10^−3^ ± 1.825 × 10^−3^, *P* = 0.0217). As such, for both 0 and 100% rewarding trials, the differences in outgoing and return peak velocities were statistically distinct. The slope of response to RPE was not found to change over the course of a block (β_RPE×Trial_ = −4.746 × 10^−3^ ± 4.770 × 10^−3^, *P* = 0.320). As in the previous experiment, we performed additional control analysis to isolate the effect of reward feedback on the return movement (fig. S10). To reiterate, even when expected reward must be experienced and learned, individuals change their behavior during the return movement in a manner consistent with RPE sign and magnitude.

#### Biomechanical effort slowed outgoing peak velocity

**Fig. 6.**
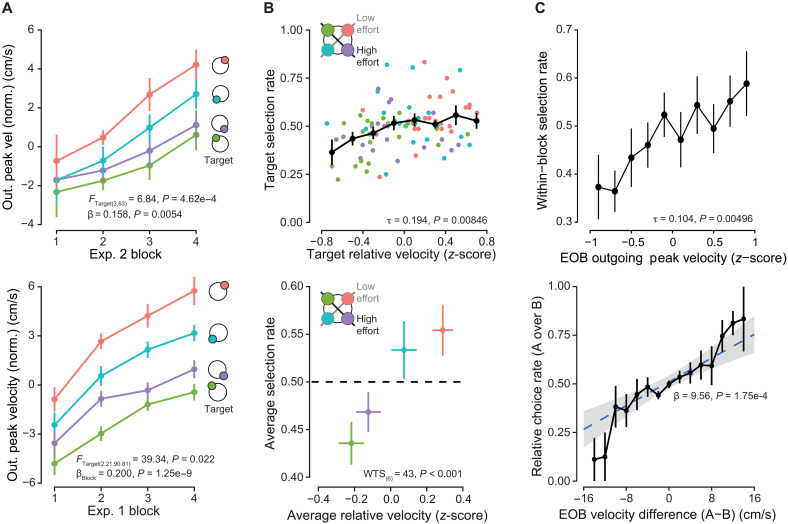
Relation between velocity, subjective effort, and learned value. (**A**) Top: In experiment 2, arm reaches toward target 1 were found to have a greater peak velocity on average, with targets 2 and 4 associated with comparatively lesser averages. Bottom: In experiment 1, the same ordering of relative peak velocities was observed. (**B**) Top: Participant-relative velocity, in single-target trials, was correlated with relative rates of selection of the different target directions. Dots are individual participants. Bottom: Averaged over all participants per target, there was evidence of a separation in relative average velocities and selection rates. (**C**) Top: Relative velocity to a given target at the end of the single-target period could predict preference. Bottom: Difference in average velocity between two targets, within the final 36 single-target trials, was continuous with relative rates of selection for those two targets. The greater the difference in velocity, the more frequently one option was chosen over the other in the proceeding six choice trials where the two targets were presented together. Dotted line and shading are linear regression model fit and SD. Points represent grand means, and error bars represent ±SEM.

Considering the effect of target direction (i.e., relative effort), on choice bias, we were also concerned whether there would be differences in the average peak velocities in the different directions. Investigating this potential effect (Fig. 6A, top), we found that each direction significantly differed in average peak outgoing velocity from the other directions (Tukey post hoc test comparison testing; table S2). In addition to the directional effect, we found that average outgoing peak velocity increased over the course of the experiment from block to block (β_Block_ = 0.158 ± 0.051, *P* = 0.0054).

We also evaluated whether this directional influence on velocity was conserved in the previous experiment (Fig. 6A, bottom). Once again, differences in average outgoing velocity across the directions were consistent with the effective mass of the arm. Peak velocity increased over the course of the experiment (β_Block_ = 0.2352 ± 0.0058, *P* = 1.25 × 10^−9^) for all targets, and post hoc multiple comparisons testing (Tukey post hoc test) revealed significant differences across all four targets (table S1).

In short, average velocity to the different target directions in both experiments reflected the relative inertia, or effective mass, of the arm when making this reaching movement. Outgoing peak velocity was greatest toward the 45° target and slowest toward the 135° target.

#### Subjective vigor response in single-target trials predicted choice

Summarizing our results, thus far, for the second experiment, we have shown how participant choices reflected subjective value, and kinematic response during single-target trials reflected the learning of relative reward frequencies. To further substantiate the relationship between vigor and preference, we examined the degree to which reach velocity during the single-target trial period could predict choices across our participant pool.

First, we analyzed whether the subjective effort bias evidenced in participant choices would also be present in the kinematic response. A comparison of the per-participant relative velocities and the target selection rates revealed a significant correlation (Kendall’s rank correlation, τ = 0.194, *P* = 0.00846; Fig. 6B, top). With multivariate analysis of variance analysis, after averaging across participants, we found significant differences between the relative velocities and selection rates across the four target directions [Fig. 6B, bottom; WTS_(6)_ = 43.165, *P* < 0.001]. Next, we questioned whether kinematic behavior during the end of the single-target period (the final 36 single-target trials) could predict later choice selections directly. To do so, we analyzed the correlation between the relative peak velocity per target and per participant and the target’s within-block selection rate (quantified as a fraction of the total 18 potential selections within the choice period). A significant correlation was found (Kendall’s rank correlation, τ = 0.104, *P* = 0.00496; Fig. 6C, top). The faster someone reached toward a target at the end of the single-target period, the more frequently they chose that option during choice trials.

As additional evidence for the relationship between outgoing peak velocity and relative selection rate, we directly compared the difference in mean velocity between two targets at the end of the single-target period and the relative rate of selection within that block. Via logistic regression analysis, we found a significant relationship between the velocity difference and rate of selection (β_ΔPV_ = 9.56 ± 2.55, *P* = 1.75 × 10^−4^; Fig. 6C, bottom). Roughly, a difference of 0.2 m/s in peak reach velocity translates to a 3:1 preferred rate of selection. Overall, we found that participant decisions varied widely across the population but could be predicted by each participant’s relative outgoing velocities on single-target trials: The greater the relative peak velocity, the more frequently that option was chosen.

### Value estimation

Reward and effort (in the form of target direction) had significant influences on outgoing velocity. Both reward and effort also affected choice preference and relative selection rates. We posit that the target characteristics of reward expectation and direction could be combined into a singular subjective value or decision variable that describes both participant-specific reach velocity and choice preference.

#### Learned value better explained single-target trial reach vigor

To examine whether this was the case and estimate this subjective decision variable, we fit a Bayesian hierarchical delta-rule learning model, similar to a Rescorla-Wagner model (*40*), to choices at the end of each block to determine the per-trial subjective value for each participant. After each trial, the updated value estimate of a target $V^{\prime T}$ is calculated

$$V_{i}^{\prime T}=V_{i}^{T}+\eta_{s}\left(R_{i}+e_{s}^{T}-V_{i}^{T}\right)$$

Here, $R_{i}$ is the reward received on the current trial, $\eta_{s}$ is the learning rate, and $e_{s}^{T}$ is the relative effort of the target, which is dependent on the direction (major or minor inertial axis). Participant-specific parameters, $\eta_{s}$ and $e_{s}^{T}$, were selected and sampled from an estimated population distribution in a hierarchical fashion. The average learning rate and effort cost were estimated as 0.182 ± 0.038 and −0.429 ± 0.141 (means ± SE), respectively.

**Fig. 7.**
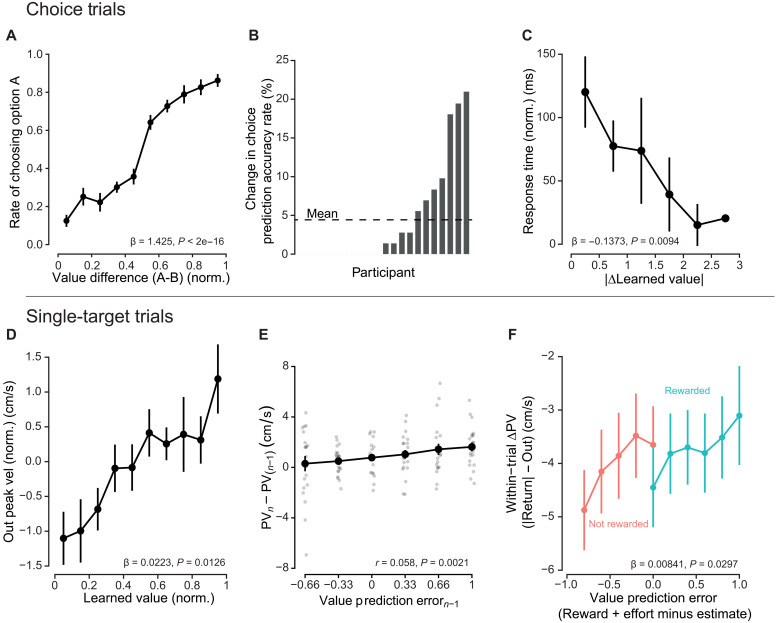
Performance of learned value model. (**A**) Individuals were sensitive to learned value differences during decisions, selecting targets with greater relative values. (**B**) Choice prediction accuracy improvement was participant specific. Average improvement was 4.42%, and some participants saw choice prediction improvements up to 20% (roughly 29 choices during the experiment). (**C**) Choice trial response time significantly decreased with difference of the presented targets’ learned values. (**D**) Outgoing peak velocity (Out peak vel) during the single-target trials significantly increased with increasing learned value. (**E**) On trials where the same target was prompted in repetition, the previous trial’s RPE was significantly correlated with the trial-to-trial change in outgoing peak velocity (PV). (**F**) VPE, taken as the difference between feedback and expected value, was also found to predict the relative difference in peak velocity between outgoing and return. Points represent grand means, and error bars represent ±SEM.

A separate logistic regression confirmed that value differences predicted choice behavior (Fig. 7A). As relative value increased, individuals were more likely to choose that option. In addition, the model-derived learned value was found to better predict choices as compared to reward expectation, with an aggregate accuracy rate of 75.4% compared to 70.98%. This finding was corroborated by a significant difference in the ROC curves between the two metrics (bootstrap test for difference in AUC: *D* = 8.054, *n* = 2000, *P* = 8.014 × 10^−16^). However, prediction improvements were highly idiosyncratic in the participant pool (Fig. 7B). Last, choice trial response time was significantly influenced by value, decreasing with increasing value difference (β_|ΔV|_ = −0.1373 ± 0.0464, *P* = 0.0094; Fig. 7C). Outgoing reach during choice trials was also influenced by the value estimate (fig. S11).

We next used this learned value estimate to predict reach peak velocity on single-target trials. There was found to be a significant relationship between value and outgoing peak velocity (GLMM; β_Value_ = 0.0223 ± 0.00895, *P* = 0.0126; Fig. 7D). The regression model including value improved in performance relative to one using *E*[*R*] [Akaike information criterion corrected (AICc) for small sample sizes: −13,479.4 versus −13,369.3, respectively]. Likewise, time to target decreased with learned value as well and better matched the data compared to reward expectation (β_Value_ = −0.0265 ± 0.007148, *P* = 0.0013; fig. S6B; AICc: −12,225.6 versus −12,157.7).

Although subjective directional efforts were incorporated into the target value, there were still significant effects of direction. Tukey post hoc test revealed significant differences between the target-specific regression factors (table S3). This implies that the difference in vigor between directions does not fully capture the resulting difference in value as shown by later choices.

We predicted, as an additional piece of evidence relating learned value and vigor, that an individual’s change in value [value prediction error (VPE)] would be correlated with the trial-to-trial change in peak velocity after a target is repeated (Fig. 7E). Calculating this correlation, we found that, as hypothesized, a greater value update led to greater velocity changes, and the relationship was significant (Pearson’s correlation, *t*_2801_ = 3.083, *r* = 0.058, *P* = 2.07 × 10^−3^). Testing to ensure that this effect was target specific, we calculated this correlation on trials where relative effort, but not direction, was repeated and found no significance (*t*_2732_ = −0.624, *r* = −0.012, *P* = 0.533). Thus, the change in outgoing vigor from one trial to the next reflected the trial-to-trial value update.

Next, we looked at the within-trial effects of VPE on vigor. The relative difference in peak velocities between the outgoing and return portions of the movement decreased with increasing VPE (β_VPE_ = 0.008414 ± 0.00370, *P* = 0.0297; Fig. 7F); the comparative velocity of the return portion of the reach was greater with more positive error. Performance in modeling the within-trial velocity difference also improved when using VPE compared to RPE (AICc: −37,118 versus −37,098.2). Unlike the RPE regression model that showed a significant effect of reward reception on average difference in return velocity (see above, Fig. 5D), the VPE model did not (β_Reward_ = −0.00441 ± 0.00296, *P* = 0.153). In addition, no significant interaction was found (β_VPE×Reward_ = −0.00510 ± 0.00298, *P* = 0.0873). In effect, the slope of response of velocity difference to VPE remained constant with or without reward feedback. Instantaneous normalized velocity difference traces also revealed an effect of VPE (fig. S12). Together, relative return velocities varied continuously with VPE with no additional significant effect of reward reception. In summary, learned value estimated from participant choice behavior was significantly reflected in movement vigor on multiple timescales: in the outgoing movement, in the change in vigor on the subsequent trial, and within the ongoing movement itself.

#### Recent history of reward led to faster movements

Having shown how target-specific learned value influenced the reach kinematics on the outgoing and return portions of the movement, we sought to investigate the potential effects of target-agnostic reward reception, independent of value estimation. We investigated the potential effect in both experiments, testing the influence of previous trial reward reception and an integrated reward history, making use of the “leaky integrator” model (see Materials and Methods) (*41*). On the basis of previous findings, we hypothesized that reward history would invigorate outgoing movement: increasing peak velocity and decrease response times.

**Fig. 8.**
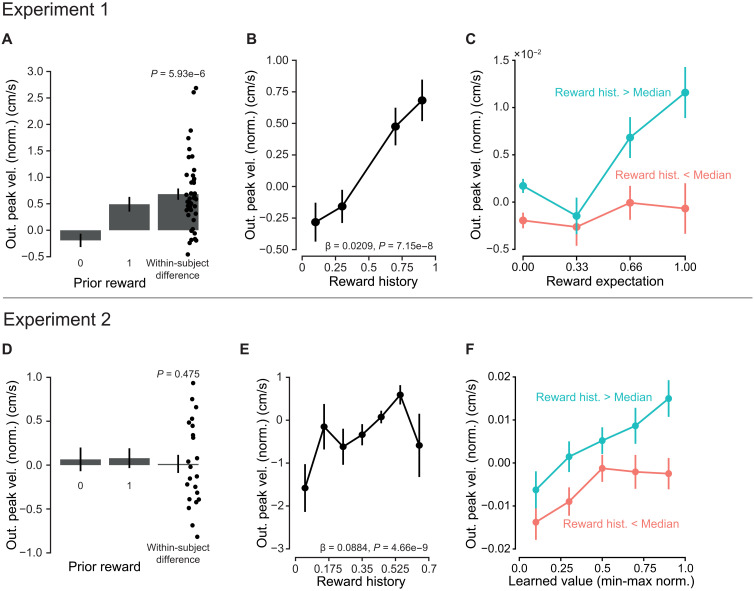
Peak velocity increased with reward history. Experiment 1: (**A**) Outgoing velocity significantly increased with prior reward reception. (**B**) With greater reward history, outgoing peak velocity increased. (**C**) Even when accounting for movements to the same reward expectation, reward history has a significant effect on outgoing peak velocity. Experiment 2: (**D**) Prior reward reception did not influence the subsequent trial velocity. (**E**) Outgoing peak velocity significantly increased with the amount of reward received in recent trial history. (**F**) Vigor of reaching movements toward targets of similar learned value was influenced by recent trial reward history. Points represent grand means, and error bars represent ±SEM. Dots represent individual participants. Out. peak vel., outgoing peak velocity.

In our first experiment, we found an effect of previous reward reception on outgoing peak velocity. Peak velocity was greater in trials following a reward than in those following no reward (β_PriorR_ = 0.0152 ± 0.003, *P* = 5.93 × 10^−6^; Fig. 8A). For our reward history model, after fitting to reaction times, the smoothing factor, α, was found to equal 0.647. Incorporating reward history into our previous regression model for outgoing peak velocity, we found that velocity significantly increased with increasing $\bar{R}$ (β_History_ = 0.0209 ± 0.00319, *P* = 7.15 × 10^−8^; Fig. 8B). Model performance also relatively improved (AICc: −85,753.7 versus −85,728.6). This model-derived reward history value was significant even when accounting for current trial reward expectations (Fig. 8C and fig. S13).

In our second experiment, unlike the first, there was no significant response caused by reward reception on the previous trial (β_PriorR_ = −0.0033 ± 0.006, *P* = 0.592; Fig. 8D). With the same reaction time-dependent likelihood maximization, we found a smoothing factor, α, equal to 0.0521. Outgoing peak velocity again significantly increased with reward history (β_Hist_ = 0.0884 ± 0.0151, *P* = 4.66 × 10^−9^; Fig. 8E and fig. S12E). Model performance when predicting velocity was also improved (AICc: *E*[*R*] + history, −36,774.7; and *E*[*R*], −36,742.4).

Last, including both learned value and reward history in a single generalized regression model significantly improved model likelihood, compared to an alternative model that excluded the reward history term (LRT: $\chi_{1}^{2}$ = 30.156, *P* = 3.987 × 10^−8^). This result strengthens our argument that both recent, target-agnostic rewards and effort-conscious valuations of specific targets influence the motivation of movement.

## DISCUSSION

Here, we demonstrated that reach vigor tracks neural correlates of learning and motivation across time scales ranging from milliseconds to minutes. Velocity was modulated by reward expectation, RPE, and reward rate, key variables that have also been associated with striatal dopaminergic fluctuations. These results point to a potential neural mechanism by which DA can provide a bridge between decision-making and movement control.

### RPE rapidly influences ongoing movements

RPE is typically examined with respect to recent reward history and current target expectation. For example, saccadic reaction times were faster or slower following a positive or negative RPE (*42*). In this study, we are the first to show that RPE can have a modulatory effect on an ongoing movement, leading to either acceleration or deceleration that aligns with the sign of the prediction error. Moreover, we find that this effect scales with the magnitude of the prediction error. The rapid reflection of RPEs in vigor may be related to vigor’s already known response to the average reward rate of the environment (*27*, *43*, *44*), which is tracked by tonic DA levels (*21*, *26*). Striatal DA levels rapidly rise and fall as a consequence of the temporal integration of phasic prediction error signals (*32*, *45*). It could be through this mechanism that we see rapid subsecond changes in vigor. Positive or negative RPEs reflect either positive or negative shifts in estimated environmental quality; thus, changes in vigor in response to errors would optimize achieved average reward rate. However, the exact relationship between spiking activity and striatal DA levels is multifaceted (*29*, *46*) and the impact that we observe on vigor is small and fleeting. This may explain why targeted optogenetic manipulations were limited or temporally restricted in their effects on vigor (*34*).

A substantial body of evidence has detailed the relationship between midbrain DA transients and action initiation (*47*). In both freely moving and head-fixed mice, phasic activity accompanied movement initiation (*34*, *48*), with its correlation to the ensuing movement’s vigor being context dependent. Likewise, many studies found significant relationships between phasic DAN activity and reward-seeking motor behaviors (*20*, *35*, *49*).

It may seem unlikely that the absence or presence of the reward feedback visual stimulus would influence ongoing velocity at subsecond speeds, ~215 ms after stimuli presentation. However, neurons in the superior colliculus, which then project to DANs within the VTA or SNc, have a response latency between 40 and 60 ms (*50*). Another study found that median response latencies of cue-elicited DAN responses within the SNc were on the order of 112 ms (*51*). Others still have found that modulation of rapid motor responses, on the order of 150 ms, is dependent on task specific visuospatial features (*52*). RPE modulation in saccade latencies was found to be affected on the order of ~150 ms (*42*). A more recent study has found that changes in direction of movement, specifically when a reach target suddenly decreases in expected value, occur, on average, ~248 ms after cue (*53*). This body of literature detailing the short-latency rapid motor response suggests that the rapid response in relative velocity that we see can be feasibly attributed to tuned sensorimotor reward-based predictions.

To generate these prediction errors, we provided individuals with stochastic reward feedback independent of motor performance. Previous research has focused on either the effect of varying reward levels (*6*, *54*, *55*) or the difference between predictable and unpredictable rewards (*3*, *56*) on the invigoration of movement. One other study reported a continuous relationship between movement response tempo (i.e., execution time) and reward-contingency belief (*57*). Our work in this study details how the reward-vigor relationship in human reaching is continuous with varying degrees of probabilistic expectation as would be predicted on the utility-maximization theoretic basis (*38*). In both explicit contexts where the reward probabilities are stated and within an implicit context where individuals must learn and experience the differences in expectations, greater reward probabilities were associated with greater average movement vigor.

### Subjective effort costs were integrated in both relative choice preference and movement vigor

In addition to the effects of within-trial reward and prediction error, we investigated the relationship between biomechanical effort, subjective choice preference, and movement vigor. Individual willingness to expend effort has been found to be idiosyncratic (*58*). We build upon this finding in our current study, showing that individuals differed in their willingness to expend biomechanical effort to accrue monetary reward and that these differences were demonstrated both in their choices and kinematic behavior during the preceding single-target trials. Specifically, the magnitude of effect of target-direction on choice preference was correlated with the magnitude of the effect on an individual’s movement vigor (Fig. 6B).

Aside from reward-mediated invigorative effects of DA, both in movement and deliberation, other research has found the neurotransmitter instrumental in overcoming costs and providing motivation in the face of effortful action (*59*–*63*), which has been shown to influence motor behavior and deliberation (*27*, *39*, *64*–*66*). However, phasic midbrain DAN activity may not necessarily code for upcoming effort value (*67*). In monkey (*Macaca mulatta*) SNc, only a minority 13% of spiking activity in a reaching task was correlated with net utility (rewards minus cost) compared to reward alone. One hypothesis is that the phasic DA response incorporates effort costs only if the reward rate, or the discounted value of future action, was meaningfully affected (*63*). Other neurotransmitters and pathways may be implicated in utility prediction. Specific serotonin receptor activation in mice was found to increase motivation and vigor alongside an increase in dorsomedial striatum extracellular DA (*68*). Thus, the effect of variable effort on the behavioral and dopaminergic response to RPE remains an open question.

A delta-rule learning model is used to estimate and quantify the trial-to-trial, subjective value of a given target, motivated by its success in modeling prediction error responses in human dopaminergic systems (*19*, *69*–*72*). We found that movement vigor reflected the learned value, which was updated from trial to trial (*43*, *57*). A critical assumption in our model is that a VPE that integrates reward and effort updates learned value, rather than solely a RPE. Effort, too, has been shown to influence neural activity in rat ventral striatum and dorsal anterior cingulate cortex (ACCd) (*73*, *74*). Likewise, human ACCd activity was found to reflect the interaction between both expected reward and expected effort (*75*). We also modeled effort as additively discounting experienced reward, rather than multiplicatively as in previous work (*76*). Additive discounting accounts for the invigorating effect of reward and aligns with the conceptual framework that the brain modulates movement vigor so as to maximize reward rate (*38*, *77*). Our approach is validated in our data where the VPE, which integrates learned reward and effort costs, has a significant effect on both the within-trial change in velocity and trial-to-trial changes (Fig. 7, E and F).

Nonetheless, an alternative approach is one where the prediction error does not incorporate the experienced target effort, which would be modeled as follows

$${\hat{R}}_{i}^{\prime T}={\hat{R}}_{i}^{T}+\eta_{s}\left(R_{i}-{\hat{R}}_{i}^{T}\right)$$

$$V_{i}^{T}={\hat{R}}_{i}^{T}+e_{s}^{T}$$

Here, learning is driven by reward prediction alone, and behavior is a result of both learned reward and already known effort. Both models were fit to choice data and compared. The first model, with learning driven from a VPE, had greater conditional coefficient of determination (*R*^2^), 0.686, compared to the alternative (0.543) when predicting participant choice. However, AICc scores show a preference toward the second model: 2890.61 compared to 2917.76. From our experimental design, the kinematic data would be equally well described with either model as effort values were constant. Ultimately, the evidence is ambiguous to state conclusively which of the feasible delta-rule models for learning target value best describes the data. Our experiment did not originally set out to test these distinctions, and so additional follow-up with the requisite design considerations would be needed.

### Environmental certainty may explain differences in reward history responses

The results highlight the differences between an experienced stochasticity and described one (*78*), although our first experiment may be better described as a hybrid protocol, as trial-to-trial feedback is still provided to reinforce the previously stated reward expectations. The use of kinematic responses, specifically peak velocity on outgoing and return portions of the movement, is a useful tool in understanding not only these differences (*79*) but also the similarities between the two environments. The effect of RPE was similar, occurring within the same time frame and showing comparable magnitudes of response. The most apparent distinction was the influence of recent reward history. In our second experiment, the found relationship between prior reward and velocity can be said to have a longer view of the past, integrating incrementally over many trials to arrive in near proximity to the reward average (0.5). The first experiment could be said to elicit more impulsive or rapid response, with the influence of trials further back in time quickly diminishing. This behavior may speak to the difference in environmental uncertainties, where average reward expectation is immediately known to the participant compared to when it must be experienced and learned over time. Previous research has found significant effects of environmental uncertainty in mice and monkey reward-learning rates (*80*), with greater uncertainty resulting in reducing update rates, which would explain differences in reward history α-coefficients between the two experiments.

### Summary

Across two experiments, we demonstrate the sensitivity of movement vigor to dopaminergic correlates of learning and motivation. As the likelihood of reward feedback increased, so, too, did vigor. Movement vigor was also rapidly updated during an ongoing movement, in proportional response to RPE. Trial-specific learned value, modeled via a delta-rule update formulation, could incorporate both rewards and subjective efforts to predict individual reaching vigor. Last, target-agnostic reward history significantly influenced vigor, even when the value expectation was matched. Together, our results highlight the link between known short-latency dopaminergic learning signals and the invigoration of movement, not only at the time of cue presentation and movement initiation but also during an ongoing movement immediately after feedback is provided.

## MATERIALS AND METHODS

### Participants

The study consisted of two experiments, each with an independent population (first: *n* = 42, *f* = 16, age = 22.3 ± 0.76; and second: *n* = 22, *f* = 16, age = 23.5 ± 0.9). All individuals were either ambidextrous or right-handed as determined by the Edinburgh Handedness Inventory survey, as well as free of upper extremity injury or self-reported neurological condition. In the first experiment, participants were compensated at a fixed rate of $10/hour with no difference depending on performance. For the second experiment, the rate of $10/hour remained with the potential of an additional $5 depending on performance (average compensation = $14.02 ± 0.13). Participants provided written and informed consent, and procedures were approved of by the University of Colorado Boulder Institutional Review Board (IRB approval no. 22-0233).

### Experimental design

Participants performed a bandit-like task using the KINARM end-point robotic arm (BKIN Technologies, ON, CA) to control a white cursor presented onscreen. From a home circle (*r* = 1 cm) at the center of a ring of radius 10 cm, individuals were instructed to make an out-and-back reaching motion to move a cursor (*r* = 0.5 cm) a cued target (*r* = 1 cm), displayed at 45°, 135°, 225°, or 315° relative to the home circle. As a consequence of this spatial orientation and biomechanical inertia of the arm, two targets (135° and 315°) had greater effective mass (*38*) compared to the other two targets (45° and 225°) and, thus, different effort costs. Target cues were displayed at a variable time interval uniformly ranging from 800 to 1000 ms after trial initiation. On reaching to the cued target, participants need not hit the prompted target exactly. If absolute angular error at the 10-cm radial distance was less than 22.5°, then the target was counted as “hit.” If individuals failed to hit the prompted target, either within the ±22.5° accuracy constraint or within 4 s, then a large red “X” appeared onscreen with an accompanying tone (400 Hz, 100-ms duration) indicating a trial failure.

Individuals were instructed that each target had a unique probability (100, 66, 33, or 0%) of providing rewarding feedback at the moment of target hit. Reward feedback consisted of a brief, high-pitched tone (880 Hz, 100-ms duration) being played, the target instantly doubling in size, changing color to yellow, and blinking intermittently (two blinks, on-off duration at 50 ms). Sample trial progression is shown in Fig. 1C. Reward probability per target changed between blocks, with a total of four blocks each consisting of 180 trials. Trial order and rewards were pseudorandomized such that, for each set of 36 trials, each target was cued nine times, and reward feedback per target was given nine, six, three, or zero times for the reward expectations of 100, 66, 33, and 0%, respectively. Individuals were not told the total number of trials to be experienced, only that the total time in the experiment would require ~1 hour.

RPE was defined as the difference between the received feedback and the presented target’s reward expectation, i.e.

$$R_{i}\in \left\{0,1\right\}$$

$$\text{RP}E_{i}=R_{i}-E\left[R_{i}^{T}\right]$$

Thus, the four target reward probabilities led to five different RPEs varying in both magnitude and sign. The probabilistic targets (33 and 66%) led to both smaller and larger, and positive and negative RPEs. The deterministic targets (0 and 100%) not only led to 0 RPE but also allowed us to compare the effects of reward feedback per se, independent of RPE.

In experiment 1, participants were explicitly told which targets were to have what expectation of reward within a block. In experiment 2, the rewards were identical to experiment 1 except that participants were not told of the target reward probabilities but rather had to learn from experience when reaching to the target when prompted. To quantify the degree to which they learned the reward probabilities, the final 36 trials of each block were “choice trials” where two targets were cued rather than a single target. No reward feedback was provided within these choice trials after a target was hit on the reach. Individuals were familiarized with the nature of these choice trials beforehand and were informed that the underlying reward frequencies remained constant within a block. Each unique choice pair (*n* = 6) was presented six times during these choice trial periods. For choice trials, we analyzed the choices as a function of expected value and expected reward. We also measured the response time, the time between target presentation and movement onset. Participants were instructed in the second experiment that additional compensation was contingent on the potential rewards received during these choice trials and, thus, were incentivized to reach toward targets with previously experienced greater expectation for reward feedback. Total bonus compensation was calculated as the sum of chosen expected reward, divided by maximum potential expected chosen reward (18.66), times $5

$$\frac{\sum_{i}E{\left[R\right]}_{\text{choice},i}}{18.66}\times \text{\$}5$$

In the first experiment, individuals were familiarized with the reaching task by completing 16 out-and-back reaches, four each to the four different targets. In the second experiment, an additional eight choice trials were provided as familiarization. Reward feedback was withheld on all familiarization trials.

### Kinematic metrics

KINARM encoders sampled hand position and velocity (*x*, *y* Cartesian coordinates) at 1000 Hz. Raw kinematics data were filtered via third-order double pass filter with cutoff of 10 Hz. Radial velocity was computed from numerical differentiation of the radial position relative to the home circle using a second-order centered finite difference. The primary metrics on each trial (both single-target trials and choice trials) were movement initiation time (reaction time), peak outgoing and return radial velocities, maximum excursion radial distance, and movement duration from onset to target hit. Reaction time, defined as the time between cue presentation and detected movement onset, was calculated by use of the MACC-based onset detection method (*81*). We also calculated a number of accuracy metrics (see the Supplementary Materials). No significant effect was observed.

### Trial exclusion criteria

For the first experiment, 1.83 ± 0.35% of trials per participant on average were excluded from kinematic analysis because of either failure to complete the trial, failing to reach the outer target ring during the initial outward reach (a “double-peak” movement), or missing the target completely (angular error was ≥22.5°). In the second experiment 7 ± 2.37% of single-target trials per participant on average were excluded from further kinematic analysis. These trials were included, however, for the purposes of calculating reward history and recorded as providing no reward feedback.

### Linear regression models

To determine the relationship between velocity, target reward, and RPE, we used generalized Gamma linear mixed models with a log-link function to estimate the relative effects of factors of interest and to account for between-participant variability. The Gamma distribution was selected after post hoc residual analysis of a Gaussian-residual assumption revealed significant heteroskedasticity and skewness. The log-link was ultimately selected after model comparisons to different potential link functions (inverse and identity). A similar analysis was used to probe the relationship between reaction time and target reward expectation.

In the first experiment, our regression models for peak velocity and reaction time was as follows

$$\begin{array}{cc} \text{Log}\left(\mu_{y}\right) & =\left(1+\text{Target}*\text{Repeat}+\text{Block}*\text{Trial}+E\left[R\right]+{\text{Prior}}_{\text{RWD}}\right)\\ & +\left(1+E\left[R\right]+\text{Target}+\text{Block}|\text{Subj}\right) \end{array}$$

with $\mu_{y}$ representing the mean of the outcome of interest, i.e., velocity or reaction time.

In addition, for the second experiment, an additional interaction term with trial was added

$$\begin{array}{cc} \text{Log}\left(\mu_{y}\right) & =\left(1+\text{Target}*\text{Repeat}+\text{Block}*\text{Trial}+E\left[R\right]+{\text{Prior}}_{\text{RWD}}\right.\\ & \left.+E\left[R\right]:\text{Trial}\right)+\left(1+E\left[R\right]+\text{Target}+\text{Block}|\text{Subj}\right) \end{array}$$

Target is treated as factored variable, and all others as continuous variables. The regression model for return peak velocity was similar, but with added terms for reward reception and prediction error while controlling for outward peak velocity

$$\begin{array}{cc} \text{Log}\left(\mu_{y}\right) & =\left(1+\text{OutPV}*\text{Target}+\text{Repeat}+\text{Trial}+\text{RPE}*\text{Reward}\right)\\ & +\left(1+\text{RPE}*\text{Reward}+\text{OutPV}+\text{Trial}|\text{Subj}\right) \end{array}$$

For velocity difference models, a Gaussian distribution was found to better approximate the residuals, so a typical LMER with an identity link function was used instead of the generalized Gamma model. All mixed regression models were fitted to restricted maximum likelihood with the lme4 and lmerTest packages for the R language (*82*, *83*), except for when model comparisons were performed with the performance R package (*84*). *P* values for mixed model regression coefficients are derived from Satterthwaite approximations. Hypothesis testing for linear combinations of regression coefficients and calculation of asymptotic chi-square test statistics was performed with the linearHypothesis function in the car R package (*85*).

### One-dimensional SPM analysis

Continuous time analysis of radial velocity was performed by one-dimensional (1D) statistical parametric mapping (*86*). Biomechanical curves are well suited to analysis by this methodology, owing to their smoothness and discrete bounds. We measured the potential effect of reward feedback on different expectation contexts, either deterministic or stochastic, with either single-sample *t* tests or two-factor analyses of variance (ANOVAs), respectively. This was done to both test for potential significant effects and when such effect may occur on instantaneous radial velocity. We focused on the time window from 150 ms before hitting the target (i.e., feedback reception) and 300 ms afterward.

Population-level inference on the effect of RPE on within-trial normalized velocity (taken as instantaneous velocity divided by the within-trial peak outgoing radial velocity) was conducted in a manner following previous work (*87*). First, regression analyzes were fitted per participant, and, then, this collection of 1D β values was submitted to a second-level single-sample *t* test.

### Learning model design

To model the trial-to-trial update process in experiment 2, we developed a Bayesian hierarchical Rescorla-Wagner learning model. In each of a block’s single-target trials, the presented target’s estimated value was updated based on this rule

$$V_{i}^{\prime T}=V_{i}^{T}+\eta_{s}\left(R_{i}+e_{s}^{T}-V_{i}^{T}\right)$$

where *T* is the presented target at 45°, 135°, 225°, or 315°; *R* is the reward feedback [0, 1], *e* is the effort cost; *s* is the participant; *i* is the trial; and η is the learning rate. Participant-specific learning rates ($\eta_{s}$) and effort costs (*e_s_*) were found via posterior maximum likelihood estimation based on choices. The full model (with priors) is characterized as follows

$$\begin{array}{c} \left(\text{Choic}e_{i}=A\right)∼\text{Bernoulli}(\theta_{i})\\ \theta_{i}=\frac{1}{1+e^{-\tau_{s}\left(V_{\text{A,i}}^{T}-V_{\text{B,i}}^{T}\right)}} \end{array}$$

$$\bar{\zeta }∼N\left(0,10\right);\bar{e}∼N\left(0,5\right);\bar{\tau }∼N\left(0,5\right)$$

$$\sigma_{\zeta }∼\Gamma \left(2,0.01\right);\sigma_{e}∼\Gamma \left(2,0.01\right);\sigma_{\tau }∼\Gamma \left(2,0.01\right)$$

$$\zeta_{s}∼N\left(\bar{\zeta },\sigma_{\zeta }\right);e_{s}∼N\left(\bar{e},\sigma_{e}\right);\tau_{s}∼N\left(\bar{\tau },\sigma_{\tau }\right)$$

$$\eta_{s}=\frac{1}{1+\text{exp}\left(-\zeta_{s}\right)}$$

In each choice trial, targets were randomly labeled as either A or B. VPE was defined as the sum of reward feedback and experienced effort minus learned value

$$\text{VP}E_{i}=R_{i}+e_{s}^{T}-V_{i}^{T}$$

Sampling for Bayesian models was performed by NUTS Hamiltonian MCMC algorithm via use of the RStan package (*88*).

### Reward history model

To investigate the effect of prior reward on excursion peak velocity, we applied an exponential moving average to reward reception history (*41*). For each single-target trial, average reward was updated with this rule

$${\bar{R}}_{i+1}=\alpha R_{i}+\left(1-\alpha \right){\bar{R}}_{i}$$

The smoothing factor, α, was fit to maximize the likelihood of a reaction time random intercept regression (*20*)

$$\begin{array}{cc} \text{Log}\left(\mu_{\text{RT}}\right) & ∼\left(1+\text{Block}+\bar{R}+\text{Repeat}+\text{Trial}*E\left[R\right]+\text{Target}\right)\\ & +\left(1|\text{Subj}\right) \end{array}$$

The effects of block number (Block), direction repetition (Repeat), trial number (Trial), reward expectation (*E*[*R*]), and factored target direction (Target) were included as control variables. We then used this model-derived reward history term to independently predict outgoing peak velocity in subsequent linear models.

## Supplementary Materials

### This PDF file includes:

Reach Accuracy Analysis Figs. S1 to S13 Tables S1 to S3
