# Supplementary material for: Rapid dopaminergic signatures in movement: Reach vigor reflects reward prediction error and learned expectation
Source: Sci Adv. 2026 Feb 27;12(9):eadz9361. doi: 10.1126/sciadv.adz9361 (PMC12947855; doi:10.1126/sciadv.adz9361)
Supplement: Supplementary file 1 — Reach Accuracy Analysis Figs. S1 to S13 Tables S1 to S3 [file sciadv.adz9361_sm.pdf]

Science Advances
AAAS

Supplementary Materials for

Rapid dopaminergic signatures in movement: Reach vigor reflects reward prediction error and learned expectation

Colin C. Korbisch and Alaa A. Ahmed

Corresponding author: Alaa A. Ahmed, alaa@colorado.edu

Sci. Adv. **12**, eadz9361 (2026)
DOI: 10.1126/sciadv.adz9361

This PDF file includes:

Reach Accuracy Analysis
Figs. S1 to S13
Tables S1 to S3

1. Effect of Reward Expectation on Reach Accuracy

To assess reach accuracy, we measured both signed and unsigned (absolute value) angular deviation relative to the target direction, as well as the signed and unsigned deviation relative to the average crossing point per subject per target direction, i.e., the normed deviation. Signed deviations provide a measure of potential bias in crossing point relative to either target center or participant average. The unsigned deviations provide a measure of crossing point variability: greater average absolute angular error indicates greater variability with respect to the crossing point.

In our first experiment, we found no significant effect of reward expectation ($p=0.774$) or target direction on the signed angular deviation, i.e., we found no evidence for systematic crossing point bias. For unsigned angular deviation, we again found no effect of reward expectation ($p=0.968$). In our normed measures of angular deviations, for both signed and unsigned, no effect of reward expectation was observed ($p=0.8024$ and $p=0.207$).

In the second experiment, neither reward expectation ($p=0.4753$) nor value ($p=0.4633$) had an observed effect on signed angular deviation. For unsigned deviation, whether testing against reward expectation or learned value, we observed no significant effects ($p=0.232$ and 0.289 respectively). As for normed measures of angular deviation, both signed and unsigned, we found no evidence for an effect of reward expectation ($p=0.262$ and $p=0.865$ respectively). Lastly, we found no evidence for an effect of value on signed and unsigned normed angular deviation ($p=0.651$ and $p=0.870$ respectively).

From our analysis across both experiments, we conclude that neither reward expectation nor learned value produced any measurable differences in reach accuracy, as quantified by mean relative crossing point angular deviation or its variability.

2. Single-Target Trial Reaction Time

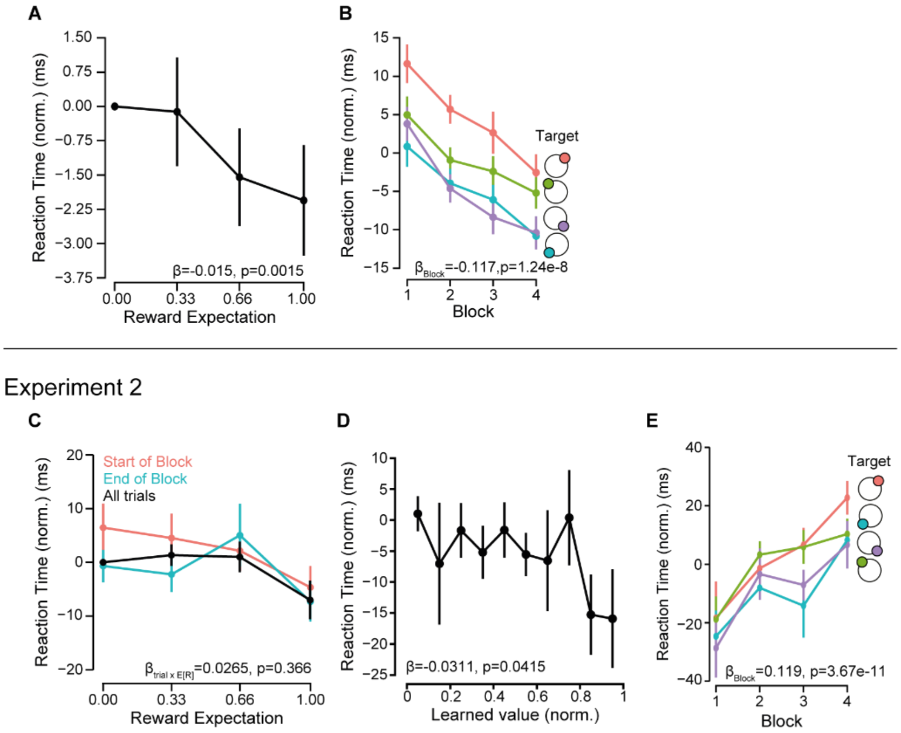

Figure S1 (**A**) In experiment 1, the time between target cue and movement onset, defined as reaction time, decreased as the cued reward expectation increased. (**B**) Reaction times, over the course of the experiment, decreased, irrespective of target direction. However, reaction times were not stratified in the same manner as was outgoing peak velocities. Post-hoc multiple comparison analysis found that movement initiation times were longer in the 45° compared to the 135° target (p adj. <0.001), equal between the 135° and 225° targets (p adj.=0.711), and longer in the 225° compared to the 315° target (p adj.=0.027). (**C**) In experiment 2, there was no systematic change in relative reaction times for the latent reward expectations over the course of the single-target period. (**D**) Single-target reaction times only marginally decreased as the estimated value for a given cued target increased. (**E**) Unlike in experiment 1, average single-target reaction times increased over the course of the experiment. Post-hoc multiple comparison analysis found that movement initiation times were indistinguishable for the 45° and 135° targets (p. adj = 0.411), longer in the 135° compared to the 225° (p adj. < 0.001), and equivalent in the 225° and 315° targets (p. adj = 0.971).

3. Experiment 1 Supplementary Results

Control Analysis of Return Movement

We performed additional control analysis to increase our certainty that it was the online reward feedback, and not other factors, that affected the ongoing movement during the return portion. For this we focused on the two stochastic targets (33% and 66%) that provided a non-zero reward prediction error. The outgoing movements for either target exhibited no difference in peak velocity based on future reward reception (for 33% trials t_{82}=0.099, p=0.9212; for 66% trials t_{82}=0.0390, p=0.969). For all reaches, the presence of reward feedback also had no effect on the extent of the reach (Gamma GLMM; β_{Reward}=-0.0485±0.054, p=0.365). Thus, for the same outgoing movement, return movements were faster when a positive, compared to a negative, reward prediction error was experienced. This effect could not be explained by differences in the extent of the reach.

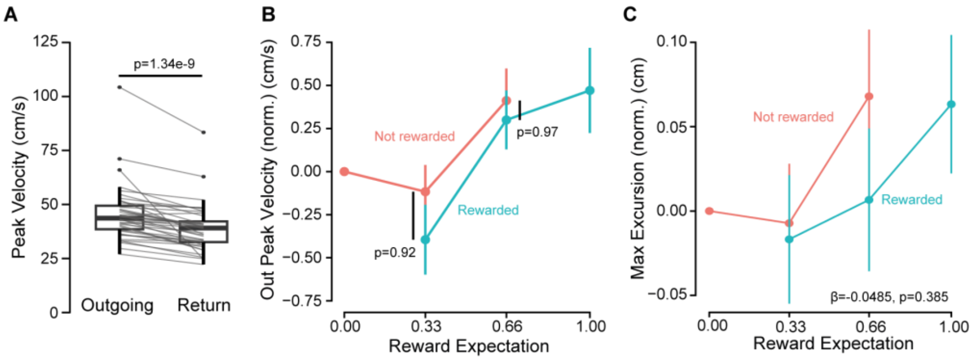

Figure S2 (**A**) Return peak velocity, on average, was less than the outgoing peak. (**B**) Outgoing peak velocity did not vary with future reward feedback. (**C**) Maximum extent of the reach did not vary with underlying reward expectation, on average, nor did it significantly change after reward feedback was received.

Effect of Reward on Return Movement

We were interested in when differences in return velocity due to reward feedback emerged. Difference traces in relative radial position, categorized by RPE condition, suggest an influence on vigor by 300 ms after receiving reward feedback. We also compared instantaneous velocity for rewarding or non-rewarding outcomes, grouped by expectation condition. There appears to be a much greater effect of reward in the return movement within stochastic conditions (E[R] = 66% or 33%) compared to deterministic reward conditions (E[R] = 0% or 100%).

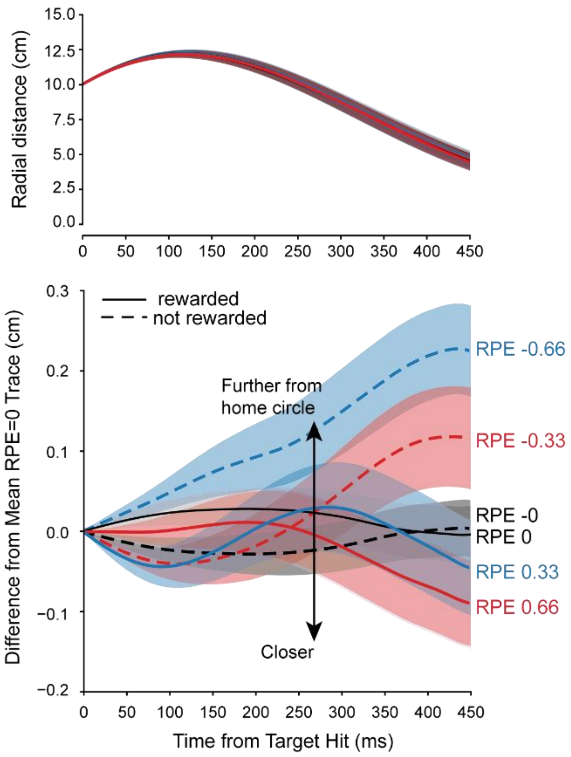

Figure S3 *Upper* Grand-average radial position traces, aligned to time of target hit, i.e., at 10 cm distance from central home circle. *Lower* Radial position difference traces were aggregated and averaged in a similar manner as in Fig. 2A. For the stochastic reward conditions, we begin to see separation in relative radial position approximately 250 ms after time of target hit. However, for the deterministic rewards, i.e. E[R] = 100% or 0%, no such distinction in radial position after reward feedback is apparent. By 450 ms after time of target hit, individuals are 2mm further away from the home circle after a -0.66 RPE compared to their average for 0% and 100% reward expectation trials. Following a +0.66 RPE, individuals are closer to the home circle by 1mm on average.

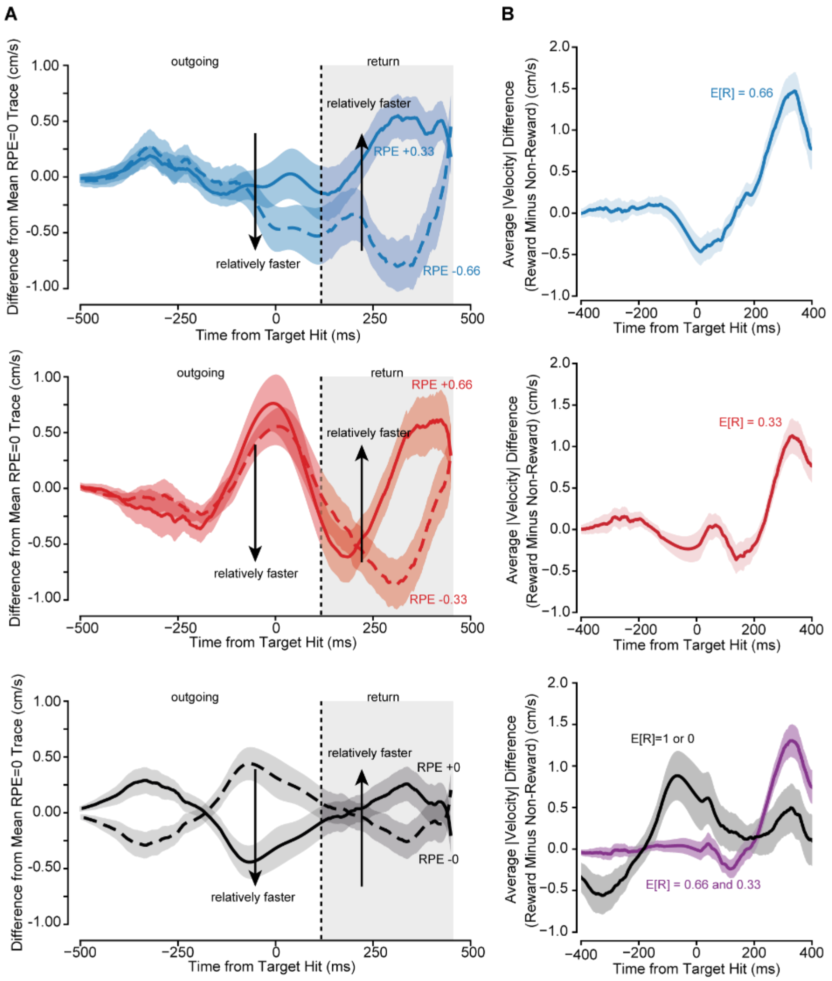

Figure S4 (**A**) Radial velocity difference trace, aligned to time of target hit, separated by reward expectation and feedback. Colors represent the different expectations and dashed or solid lines represent the absence or presence of reward feedback, respectively. After reward feedback, relative velocities for the same initial expectation differed: more positive RPEs lead to relatively greater return velocities, while negative RPEs led to more negative, i.e. slower, return velocities. Data were subtracted and averaged in a manner similar to figure 2A, but difference was taken to the average of all trials with reward expectation of 1 or 0, rather than only 0. (**B**) Instead of taking the difference to the subject-average for RPE=0 condition, absolute radial velocity data were subtracted from the per-subject average for rewarding and non-rewarding trials for a given reward condition. We see reward feedback produced marked changes in relative velocities approximately 220 ms, on average, after feedback was received. Notably, we do not see a similar magnitude of change for the RPE=0 conditions (bottom).

Return Movement SPM Analysis

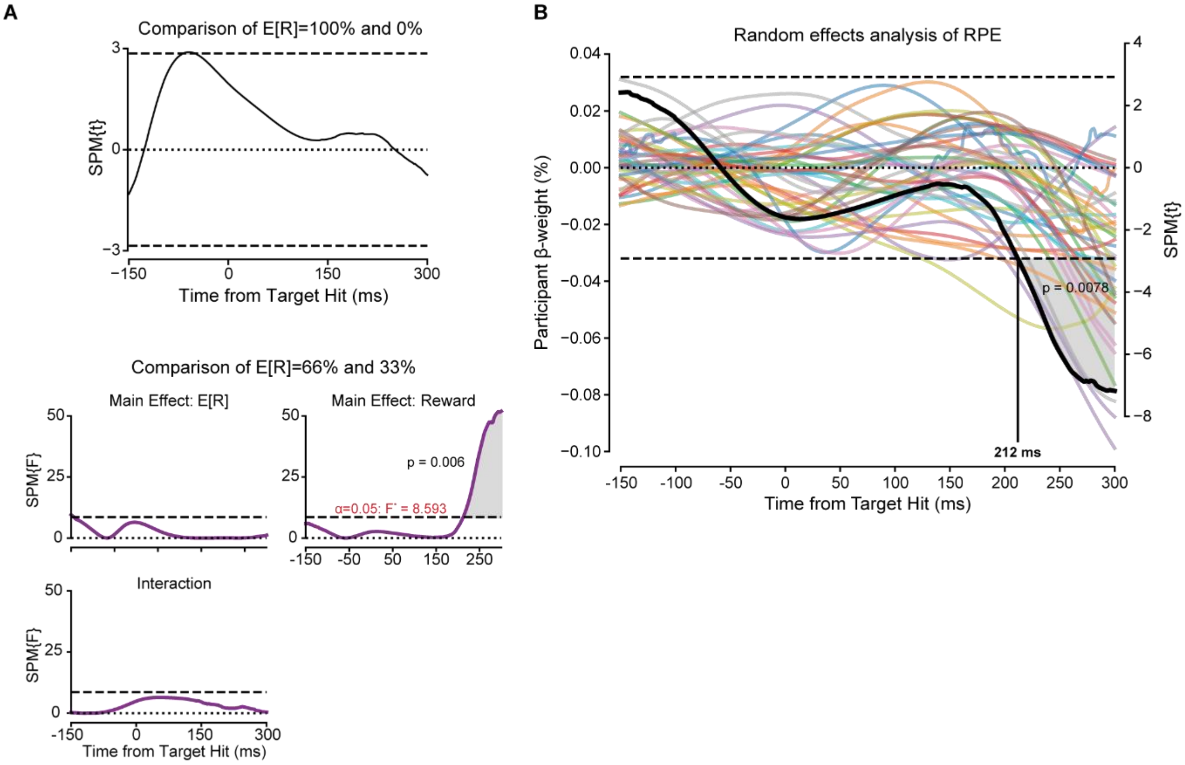

Figure S5 (**A**) ANOVA SPM results comparing the deterministic and stochastic reward conditions. Single factor analysis reveals no effect of reward feedback in the deterministic case in the time after target hit. For stochastic rewards, 2-factor analysis reveals a significant effect of reward feedback at ~200ms after feedback was received. (**B**) Hierarchical SPM regression analysis reveals a consistent and significant slope effect of RPE on relative instantaneous velocity 212 ms after target hit.

4. Experiment 2 Supplementary Results

Choice Trial Kinematics

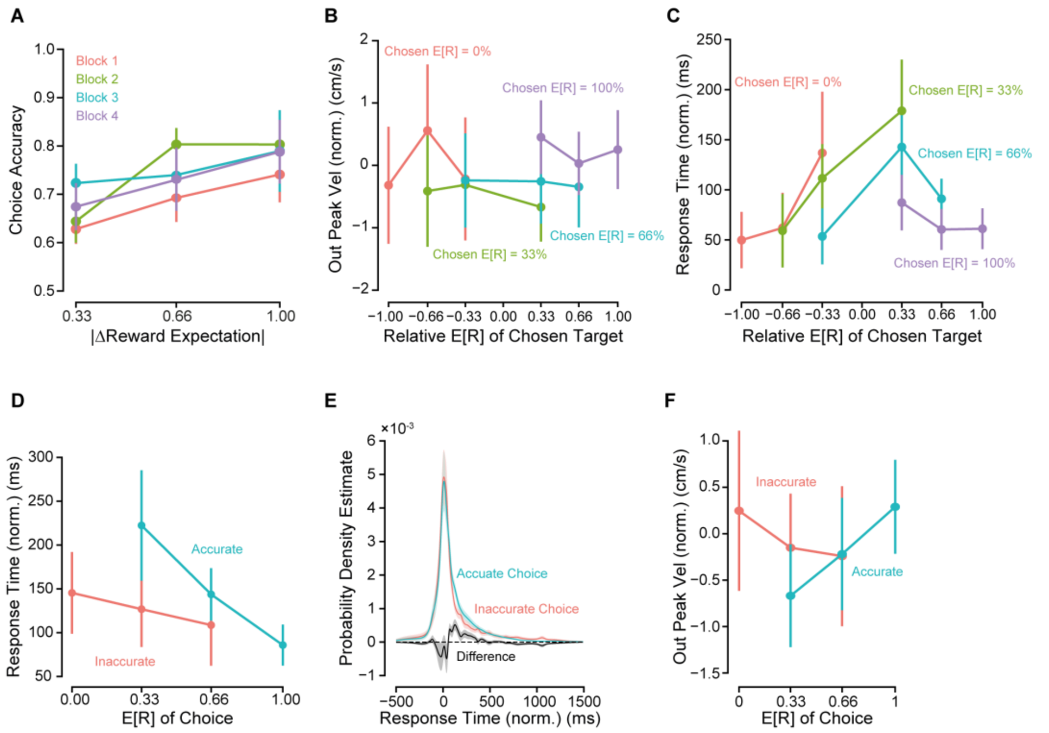

Figure S6 (A) Subject-average choice accuracy, defined as the rate of choosing the more frequently rewarding of the two options presented, increased as the reward difference increased, but did not significantly vary across experimental blocks. (**B**) Outgoing peak velocity during choice trials varied with the expected reward of the choice, but not the relative reward value. (**C**) Response times during choice trials varied with both relative and expected reward amounts. (**D**) Movement onset time during choice trials (response time) decreased as the expected reward of the chosen option decreased. This effect, however, was dependent on choice accuracy, as the slope of response was less for inaccurate choices. (**E**) Distributional analysis of subject choice response times reveals that accurate choices had longer response times compared to inaccurate choices. (**F**) Peak velocity response with respect to underlying reward expectation appeared dependent on relative choice accuracy.

Reward and Effort Sensitivity

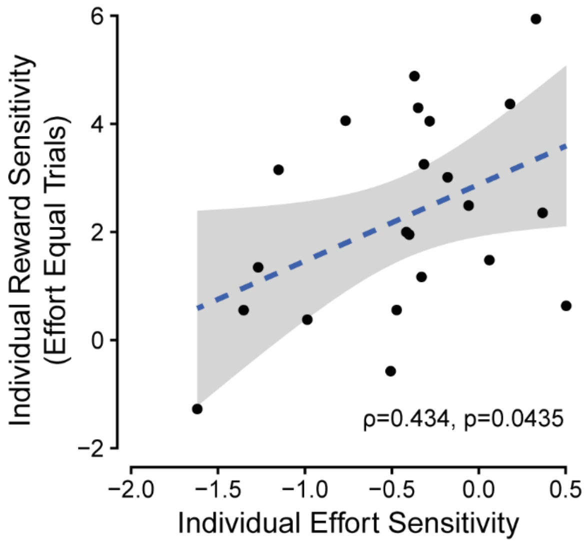

Figure S7 To examine individual relative sensitivities to reward and effort differences in choice trials, we compared random-slope responses to effort differences for all choice trials to random-slopes for reward differences within the subset of choice trials where efforts were matched (i.e. the 45° and 225° targets, or the 135° and 315° targets). Comparing these values, we found a significant correlation between the two (ρ=0.434, p=0.04346). The more effort sensitive individuals (more negative values) were found to be less reward sensitive (more negative values).

Outgoing Movement

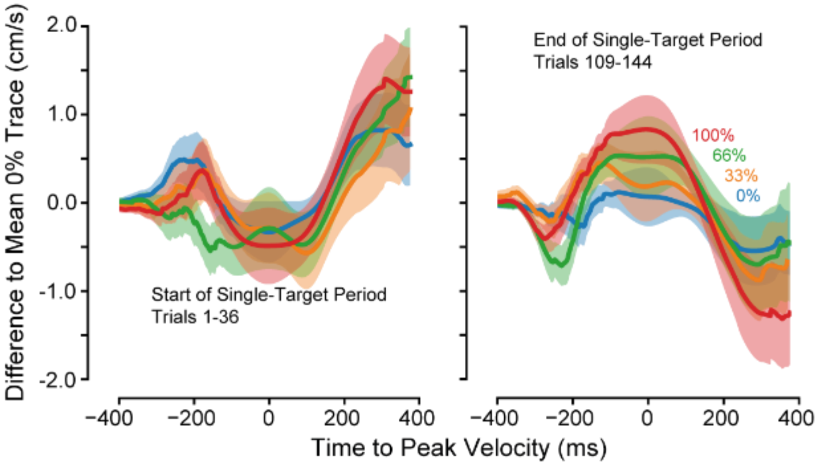

Figure S8 Velocity difference traces were calculated in the same manner as in Figure 2, with the difference taken to the within-subject average velocity trace for the 0% reward condition across the entire block. *Left* In the first 36 trials of the single-target period, there was little if any differentiation between trajectories towards the four different reward expectations. *Right* By the final 36 trials, peak velocity was differentiated between the 4 different reward expectations.

Return Movement SPM Analysis

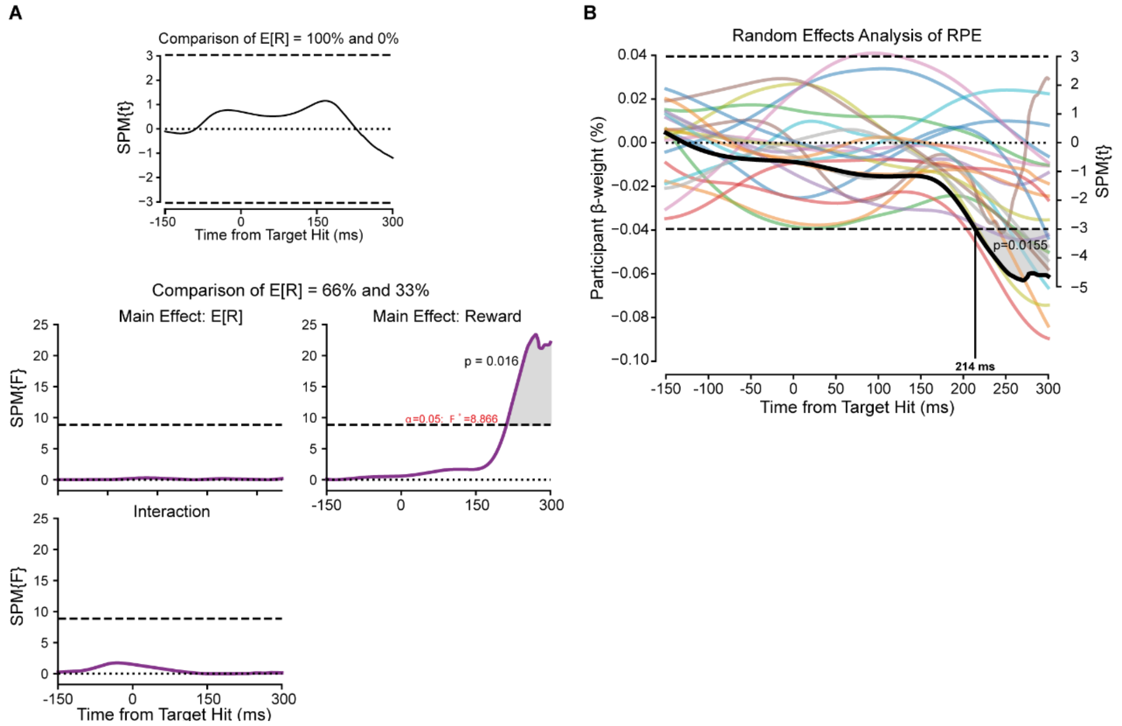

Figure S9 (**A**) Multiple SPM ANOVA analysis, as in experiment 1, found a significant effect of reward feedback within the stochastic reward trials (E[R] = 33% or 66%). (**B**) Hierarchical SPM regression analysis found a significant effect of reward prediction error on instantaneous radial velocity beginning 214 ms after reward feedback was provided.

Control Analysis of Return Movement

As before, we controlled for potential differences in average peak velocity between the outgoing and return portions of the movement, effect of future reward, as well as maximum excursion. We additionally tested whether the effects of RPE on single-target trial return movements were spurious by leveraging the fact that reward feedback is withheld during choice trials and thus providing no prediction error. To test and generate a feasible RPE value, we randomly simulated reward prediction errors for each choice trial depending on participants' selections and underlying reward frequency. After each of the 1000 simulations performed, we regressed this value against the within-trial difference in peak velocities. Following this process, only 85 of the 1000 models produced nonzero regression coefficients with significance ≤0.05. Mean p-value for β_{RPE} equaled 0.263 [Wald 95% CI: 0.2357, 0.2903]. In effect, no strong evidence of hypothetical feedback during choice trials was found to predict changes in peak velocity.

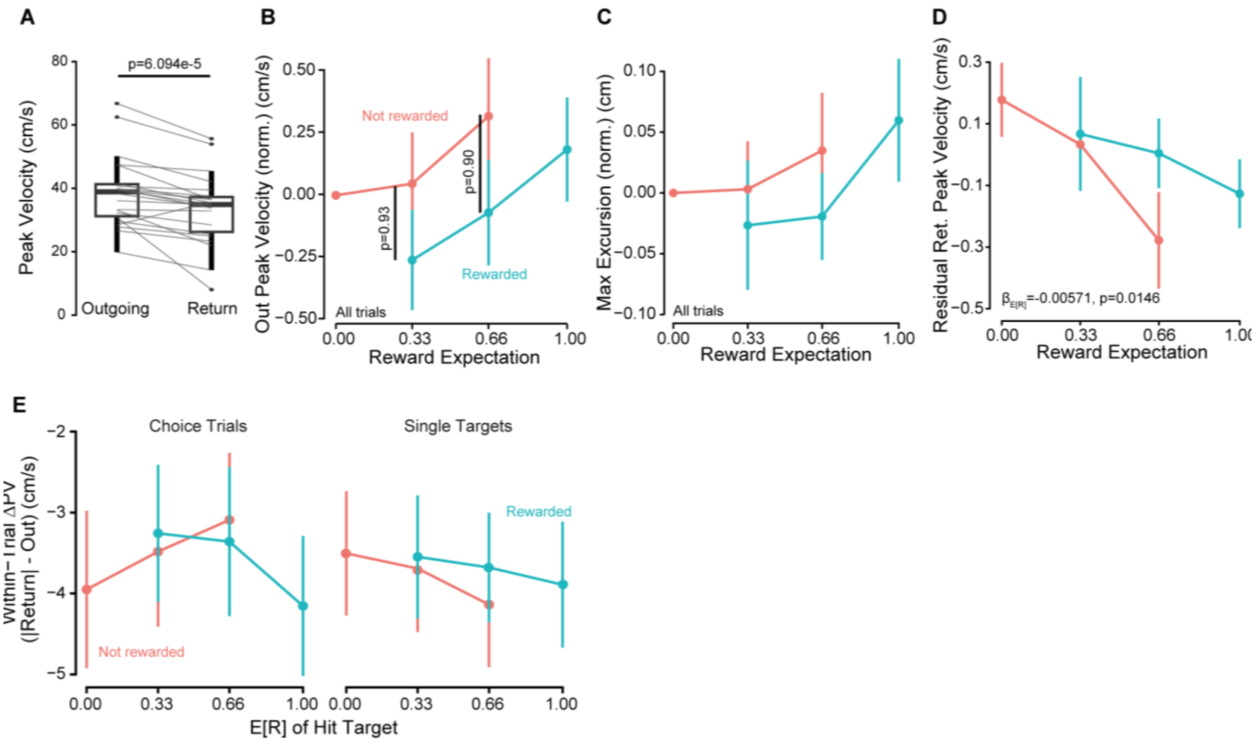

Figure S10 (**A**) Return peak was, on average, less than the outgoing peak velocity, as in the first experiment. (**B**) Effect of reward feedback or RPE could not be accounted for by differences in outgoing peak velocity. (**C**) Likewise, maximum excursion was indifferent to the reception of reward feedback (β=-3.79e-3, p=0.088). (**D**) Residualized analysis of return peak velocity revealed, as in experiment 1, a significant effect of reward expectation on the return peak. (**E**) Control analysis for the effect of reward feedback on within-trial difference in return peak velocity.

Additional Analyses

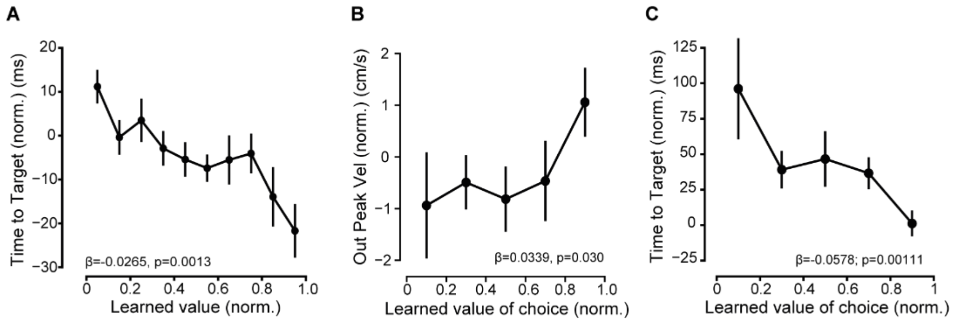

Figure S11 (**A**) In single target trials, the time to target significantly decreased as the value estimate of a given target option increased. (**B**) Within choice trials, outgoing peak velocity increased with increasing subjective, learned value of the chosen target. (**C**) Similarly, in choice trials, time to target decreased as the chosen value increased.

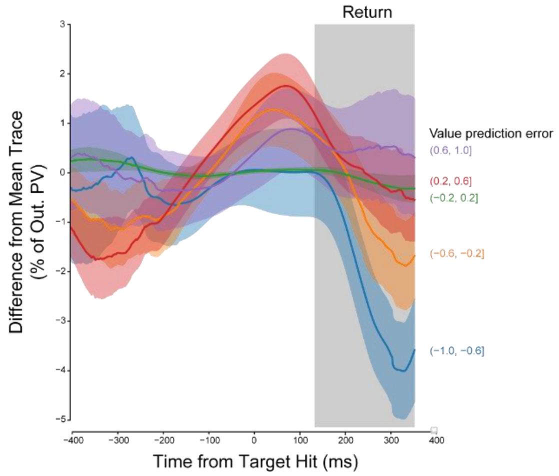

Figure S12 Velocity difference traces, binned by experienced value prediction error. Data were normalized in a manner similar to Figure 2, with differences taken to the within-participant average instantaneous velocity for trials with ±0.2 value prediction error, as well as the same experienced reward (0 or 1). Relative velocities on the return portion of the reach appear less consistently striated compared to the reward prediction error results (Figure 5), likely due to the unbalanced trial and target counts per prediction error bin across the participant population.

5. Reward History

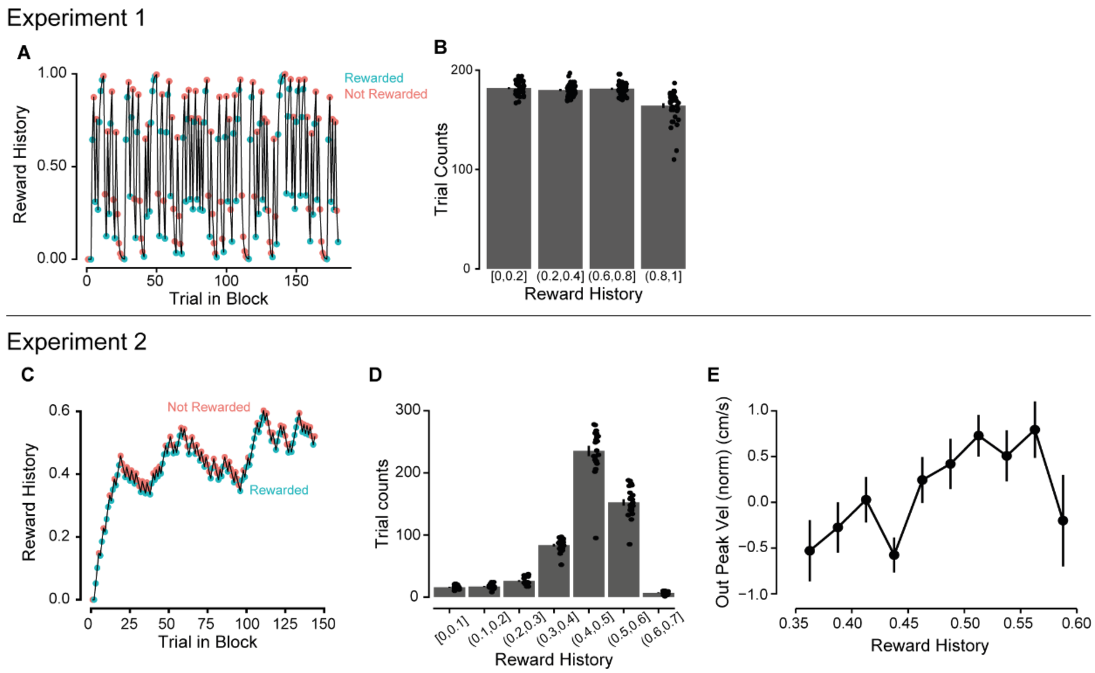

Figure S13 A) Example reward history values for a single subject and block in the first experiment. B) Averaged trial counts for reward history bins across the subject population in experiment 1. C) Example reward history values for a single subject and block in experiment 2. D) Averaged trial counts for reward history bins in experiment 2. Due to the differences in alpha-coefficient (leaky-integration rate), history values were much less uniform and more concentrated on the expectation mean as compared to experiment 1. E) Highlighting only the final 108 single-target trials, we still see a linear relationship between the reward history value and relative outgoing peak velocity, indicating that this history effect is not driven merely by the initial 36 trials (see *C*).

6. Effect of Target Direction on Peak Velocity

Supplementary Table S1. Experiment 1: Post-hoc multiple comparison testing for differences in excursion peak velocity between the four target directions. Significant adjusted p-values for target velocity comparisons are in **bold**.

PV Comparison	Mean Difference Estimate	Std. Error	z value	Adj. $P_r(>\|z\|)$
$PV_{135} - PV_{45}$	-0.05206	0.005361	-9.711	**< 0.001**
$PV_{225} - PV_{45}$	-0.02084	0.006761	-3.083	**0.01025**
$PV_{315} - PV_{45}$	-0.03804	0.006197	-6.138	**< 0.001**
$PV_{225} - PV_{135}$	0.03122	0.004717	6.618	**< 0.001**
$PV_{315} - PV_{135}$	0.014029	0.003943	3.557	**0.00212**
$PV_{315} - PV_{225}$	-0.01719	0.004047	-4.248	**< 0.001**

Supplementary Table S2. Experiment 2: Post-hoc multiple comparison testing for differences in outgoing peak velocity between the four target directions. Significant adjusted p-values for target velocity comparisons are in **bold**.

PV Comparison	Mean Difference Estimate	Std. Error	z value	Adj. $P_r(>\|z\|)$
$PV_{135} - PV_{45}$	-0.06669	0.003946	-16.9	**< 0.001**
$PV_{225} - PV_{45}$	-0.03109	0.003969	-7.834	**< 0.001**
$PV_{315} - PV_{45}$	-0.05429	0.003958	-13.716	**< 0.001**
$PV_{225} - PV_{135}$	0.035599	0.003961	8.987	**< 0.001**
$PV_{315} - PV_{135}$	0.012404	0.003941	3.148	**0.00876**
$PV_{315} - PV_{225}$	-0.0232	0.003971	-5.841	**< 0.001**

Supplementary Table S3. Experiment 2: Post-hoc multiple comparison testing for differences in outgoing peak velocity between the four target directions after application of mixed regression to model the effect of learned value that integrated both reward and effort. Significant adjusted p-values for target velocity comparisons are in **bold**.

PV Comparison	Mean Difference Estimate	Std. Error	z value	Adj. $P_r(>\|z\|)$
$PV_{135} - PV_{45}$	-0.0663	0.01460	-4.542	**3.135e-5**
$PV_{225} - PV_{45}$	-0.0306	0.0129	-2.380	0.0776
$PV_{315} - PV_{45}$	-0.0546	0.0131	-4.174	**1.859e-4**
$PV_{225} - PV_{135}$	0.0357	0.0186	1.920	0.2128
$PV_{315} - PV_{135}$	0.0117	0.0150	0.783	0.8572
$PV_{315} - PV_{225}$	-0.0240	0.0158	-1.522	0.4145
